# Supplementary material for: Task sharing for increasing access to obstetric ultrasonography: a formative qualitative study of nurse-led scanning with telemedicine review in Kenya
Source: Oxf Open Digit Health. 2024 Aug 28;2:oqae037. doi: 10.1093/oodh/oqae037 (PMC11932403; doi:10.1093/oodh/oqae037)
Supplement: Appendix_2_Code_Tree_Sonomobile_oqae037 [file Appendix_2_Code_Tree_Sonomobile_oqae037.docx]

**Technology (5)**

**Technology Challenges (5).**

**Improved capacity and decision making for maternal care 48.**

**Improved maternal care 6**

**Averted complications 3**

**Informs ANC 1**

**Reduced referral cases 1**

**Behaviour change 1**

**Quality Images 3**

**Nurses’ flexibility 1**

**Fast Results 5**

**Improved capacity and decision making for maternal care (70)**

**Accurate and reliable reports 5**

**Gender prediction 7**

**Reliability 4**

**Planning for pregnancy 1**

**Accurate and reliable results 3**

**Planning for pregnancy eventualities 2**

**Trust 1**

**Errors in measurement 2**

**Inadequate calibration of software 1**

**Quality Assurance 5**

**Accurate and reliable reports (31)**

**Mothers spread awareness/ Sensitization on service 8.**

**Lack of awareness 2**

**Myths and misconception 1**

**Demystified myths 1**

**Health education 5**

**Improved awareness/ Sensitization 9**

**Awareness (26)**

**Patient Centered Care 6**

**Client satisfaction 2**

**Friendly staff 2**

**Close contact with mothers 1**

**Interactive sessions 1**

**Received ultrasound image 1.**

**Message reminders 1**

**Unavailability of printed images 4**

**Images sent to the phone 1.**

**No printed reports 1**

**Patient Centered Care (20)**

**Limited access 2**

**Services available for few days 2**

**Accessibility 1**

**Predictable schedule 1**

**Easily accessible 1**

**Paradigm shift 1**

**Improved access to obstetric imaging. 14**

**Increased ANC uptake 1**

**Access to Obstetric Scans (26)**

**Limited capacity of the machines 7**

**Delayed process 1**

**Limited integration with analogue phones 1**

**Delays in service delivery 5**

**User friendly outputs 6**

**Low quality Images 3**

**Client complains 1**

**Capacity of Machines and Quality of Images (24)**

**Stakeholder engagement 2**

**Strategic partnerships 1**

**Implemented in similar countries (Precedence) 1**

**Referral systems 4**

**Questioned legitimacy 9.**

**No regulations 1**

**Competing interest with sonographers 1**

**Stakeholder engagement for legitimacy (19)**

**Affordability 26**

**Willingness to pay 4.**

**High costs of training 2**

**Limited resources 1**

**Affordability (33)**

**Staff released/ terminated 1.**

**High workload 4**

**Few sonographers 1**

**Staffing and Workload (6)**

**Reduced client turn-out 6.**

**Demand for Services (6)**

**Limited scope/ capacity of the Nurses 7**

**Repeat scans 1**

**Standardization of training 2**

**More capacity/ training of nurses 2**

**Centralized coordination 1**

**Complementarity between nurses and sonographers 8**

**Clarity of roles 1**

**Capacity of Nurses, Training & Complementarity (21)**
